# Nitric Oxide-Releasing Polymeric Materials for Antimicrobial Applications: A Review

**DOI:** 10.3390/antiox8110556

**Published:** 2019-11-15

**Authors:** Fan Rong, Yizhang Tang, Tengjiao Wang, Tao Feng, Jiang Song, Peng Li, Wei Huang

**Affiliations:** 1 Xi’an Institute of Flexible Electronics & Xi’an Institute of Biomedical Materials & Engineering, Northwestern Polytechnical University, 127 West Youyi Road, Xi’an 710072, Shaanxi, China; 2 Department of Applied Chemistry, School of Natural and Applied Science, Northwestern Polytechnical University, 127 West Youyi Road, Xi’an 710072, Shaanxi, China; 3 School of Electronics & Information, Northwestern Polytechnical University, 127 West Youyi Road, Xi’an 710072, Shaanxi, China

**Keywords:** nitric oxide, NO releasing polymer, anti-bacterial, anti-biofilm, biomedical device

## Abstract

Polymeric materials releasing nitric oxide have attracted significant attention for therapeutic use in recent years. As one of the gaseous signaling agents in eukaryotic cells, endogenously generated nitric oxide (NO) is also capable of regulating the behavior of bacteria as well as biofilm formation in many metabolic pathways. To overcome the drawbacks caused by the radical nature of NO, synthetic or natural polymers bearing NO releasing moiety have been prepared as nano-sized materials, coatings, and hydrogels. To successfully design these materials, the amount of NO released within a certain duration, the targeted pathogens and the trigger mechanisms upon external stimulation with light, temperature, and chemicals should be taken into consideration. Meanwhile, NO donors like *S*-nitrosothiols (RSNOs) and *N*-diazeniumdiolates (NONOates) have been widely utilized for developing antimicrobial polymeric agents through polymer-NO donor conjugation or physical encapsulation. In addition, antimicrobial materials with visible light responsive NO donor are also reported as strong and physiological friendly tools for rapid bacterial clearance. This review highlights approaches to delivery NO from different types of polymeric materials for combating diseases caused by pathogenic bacteria, which hopefully can inspire researchers facing common challenges in the coming ‘post-antibiotic’ era.

## 1. Introduction

In the 1940s, penicillin—the first antibiotic discovered in the world—was clinically applied, saving the lives of many soldiers suffering from bacterial infections during World War Ⅱ. Since then, various antibiotics have been developed and widely used in clinical anti-infection treatment. However, antibiotics have been abused in recent years, resulting in the emergence and ubiquity of drug-resistant bacteria [1], and several multi-drug resistant bacteria are significantly resistant to almost all available antibiotics in clinical practices. There are two main reasons for the emergence of multi-drug resistant bacteria. The first is that some bacteria such as *Mycobacterium tuberculosis* can prevent the binding of antibiotics by changing the protein structure on the surface of their cell membranes [2]. The other reason is the formation of bacterial biofilms. Bacterial biofilm is a structured bacterial community enclosed in a self-produced polymeric matrix that mainly consists of polysaccharides, proteins, nucleic acids, and lipids, and usually adhere to a surface [3,4]. Biofilms offer a much more robust defense mechanism than individual planktonic bacteria [5]. Due to the reasons mentioned above, it has been difficult for antibiotics to have the same bactericidal effect as before. Serious infections caused by multi-drug resistant bacteria have become a major global health care problem in the 21st century. They are usually more severe than conventional bacterial infections, and require more costs for treatments. Therefore, the world may even enter a “post-antibiotic era” [6] (proposed by Alfonso in 2005), which undoubtedly constitutes a “vicious circle” disaster [7]. Facing these challenges, researchers are actively exploring new classes of antibacterial agents and effective therapies to deal with the threaten caused by multi-drug resistant bacteria. Antibacterial properties of several advanced antimicrobial agents like inorganic antimicrobial agents (copper, silver, zinc, etc.), synthetic antimicrobial agents such as polycations (quaternary ammonium salts, quaternary phosphonium salts, etc.) and natural biological antimicrobial agents such as chitosan and antibacterial peptides [8,9,10,11,12] have been quite thoroughly investigated. Furthermore, nano-sized materials with specific structures (brush, star, and branch) containing antibacterial moieties have been proven to be effective in antibacterial application. In addition, novel antibacterial therapies including photothermal therapy, photodynamic therapy [13], and phage therapy are attracting interest. In recent years, using gaseous signaling agents to combat multi-drug resistant bacteria has gained a lot of attention, especially in the research of NO releasing antimicrobial polymeric materials.

For a long time, nitric oxide (NO) has been considered only an atmospheric pollutant. In fact, NO plays important roles in the physiological activities of animals [14], plants [15], and microorganisms [16]. NO is an endogenously synthesized diatomic molecule with a radical character that exists in various tissues and cells of the human body and is widely involved in the regulation of many physiological and pathological processes [17]. In the 1980s, Murad, Furchgott, and Ignarro discovered that NO is an endothelium-derived relaxing factor which can dilate blood vessels, thus regulating blood pressure [18,19,20]. This research aroused great interest among researchers, and in their later researches NO was identified to be a vital signaling molecule for the regulation of many physiological activities in the human body. NO was selected as Science Magazine’s *Molecule of the Year* in 1992 [21]. In October 1998, Furchgott, Ignarro, and Muard won the Nobel Prize in the field of physiology and medicine for their outstanding work in NO research. Since then, the enthusiasm for researches on NO has almost reached the peak, and it has been increasingly valued in biology and medicine. To sum up, the roles played by endogenous NO in the human body are as follows: (i) NO is an endothelium-derived relaxing factor that relaxes vascular smooth muscle and prevents platelet aggregation [22,23]; (ii) NO is a reverse messenger of nerve conduction and plays an important role in the process of learning and memorization [24,25]; (iii) when activated upon phagocytosis and stimulation, macrophages release NO as toxic molecules that kill foreign invading microorganisms and tumor cells [26,27,28,29]; (iv) as a free radical, NO can damage normal cells, which plays an important role in myocardial and brain ischemia–reperfusion injuries [30,31]; and (v) NO can regulate the inflammatory reaction and cell proliferation processes, which are key to the wound healing process [32,33,34].

Recent research has shown that exogenous NO has a therapeutic effect on human diseases at appropriate concentrations. For example, NO can synergistically enhance the anticancer ability of some anticancer medicines [35,36,37]. Additionally, it has been identified that exogenous NO can actively inhibit bacteria proliferation or directly eliminate bacteria in a relatively high concentration. However, its radical nature, uncontrolled manner and toxicity to humans prevent the direct application of NO. To utilize the antibacterial property of NO in a controlled way, NO donors releasing NO under specific conditions especially physiologically friendly visible light have been explored. Furthermore, NO releasing moieties were incorporated with different scaffolds to be applied in many conditions. Polymeric materials can be easily modified and made into various structures, representing powerful scaffolds for NO loading, which can also enhance the stability of NO donors and modulate the NO release profiles [38]. Many polymeric NO releasing materials have been developed for antibacterial application, which demonstrate more significant antibacterial effects than conventional antimicrobial agents while avoiding resistance [39], especially on bacteria in the form of biofilms. In this review, we briefly overview the antibacterial mechanism and detecting methods of NO, and reviewed the recent research of polymeric NO releasing materials for antibacterial applications, highlighting their controlled release property, anti-biofilm ability, and potential for future use in indwelling medical devices.

## 2. Antibacterial Mechanism of NO 

Since its discovery as a signaling medium, NO has been associated with numerous physiological processes in the immune, cardiovascular, and nervous systems [40,41]. In the past few years, the antibacterial property of NO has attracted the attention of many researchers, and the antibacterial mechanism of NO has become clear.

**Figure 1:**
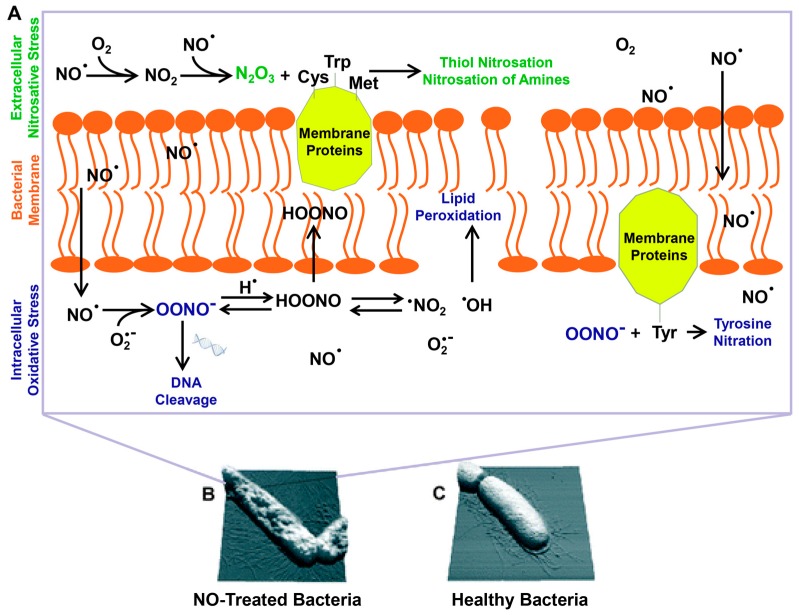
The multiple antimicrobial mechanisms of NO and its byproducts (**A**). Bacteria on NO releasing surfaces (**B**) and on controlled surfaces (**C**). Reproduced with permission from [47]. Copyright 2012, Royal Society of Chemistry.

The antibacterial property of NO is dose-dependent. At low concentrations (usually below 1μM), NO shows limited bacterial-killing effect. Similar to eukaryotic cell, NO is an important biosignal molecule in bacteria [16,42]. Bacteria also have nitric oxide synthases (bacterial NOSs, bNOSs), but they lack an essential reductase domain. Thus, the generation of NO by bNOS requires the help of eukaryotic reductases in vivo [43,44]. Research has shown that NO generated by bNOS can protect bacteria from immune oxidative bursts [45] and increase bacterial antibiotic resistance [44]. At high concentrations, NO shows bacteria-killing effects, and a widely recognized antibacterial mechanism of NO is described below (Figure 1). As a free radical, NO is unstable in cells and can easily react with oxygen or reactive oxygen intermediates such as superoxide (O_2_^−^) and hydrogen peroxide (H_2_O_2_) to form products with highly oxidizing activities including peroxynitrite (ONOO^−^), nitrogen dioxide (NO_2_), dinitrogen tetroxide (N_2_O_3_), and so on. These reactive species can interact with proteins through reactive thiols, amines, heme groups, iron–sulfur clusters and other groups such as phenol or aromatic amino acid residues [46]. For example, N_2_O_3_ and ONOO^−^ can oxidize membrane proteins at different sites, through nitrosation of reactive thiols and amines, or nitration of tyrosine [47]. NO can react with metalloenzymes, resulting in iron depletion. It can also react with free thiol groups. Both reactions result in the inactivation of metabolic enzymes [48]. Reactive species can also target DNA [49], causing deamination and oxidative damages, and finally lead to DNA cleavage. Furthermore, they can also induce lipid peroxidation to damage cell membranes [50]. The multifactorial damage to bacteria results in dysfunction and ultimately cell death [47,51]. Recent research has shown that NO can induce the dispersal of bacterial biofilms, the mechanism of which will be discussed later in this review. 

## 3. Methods for Detecting NO Release

**Figure 2:**
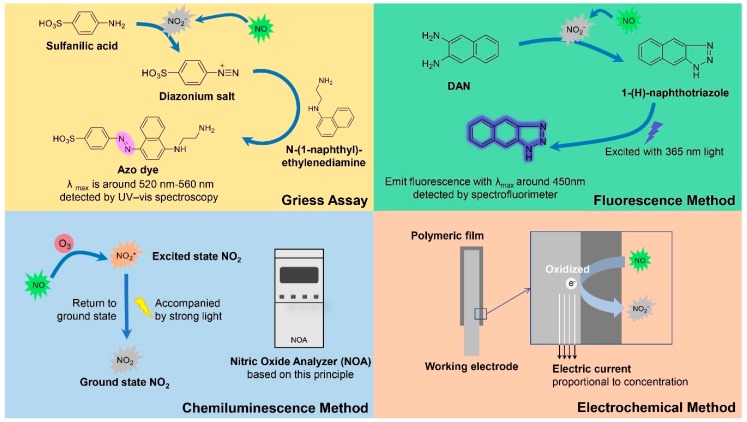
Mechanisms of four methods for detecting NO release.

Since the antibacterial ability of NO is dose-dependent, the release behavior of NO releasing polymeric materials should be the primary issue in the following researches once the materials are prepared. For example, the amount of NO release should be adequate to kill bacteria, and the release time of NO should be carefully detected when long-term application is required. Furthermore, a real-time detection of NO release is needed when we evaluate the light-responsive property of photo-triggered NO releasing polymers and their stability without light irradiation. Therefore, an appropriate method for detecting NO release is a key factor in the research of NO releasing antibacterial polymeric materials. Additionally, different NO detecting methods have different sensitivity and detection limits, which are suitable for different conditions. Herein, we introduce four commonly used methods for NO detection (Figure 2), focusing on their detecting mechanism, advantages, application conditions, and limits in antibacterial researches.

### 3.1. Griess Assay

Griess assay is commonly used as a method for detecting NO_2_^−^ and NO, which is based on Griess reaction developed by Griess in 1879 [52,53]. Griess Reagent, containing equal part of *N*-(1-naphthyl)ethylenediamine and sulfanilic acid, is used in this assay. The mechanism of Griess reagent can be elaborated as follows: NO is easily oxidized to NO_2_^−^ in vivo or in aqueous solution, and NO_2_^−^ can react with sulfanilic acid to form a diazonium salt in acid media. This diazonium salt then reacts with *N*-(1-naphthyl)ethylenediamine to form a purple colored azo dye, which can be quantitatively determined at about 520–560 nm by UV-vis spectroscopy [54]. Since Griess assay can only detect NO_2_^−^ but not further oxidized NO_3_^−^, the NO_3_^−^ must be reduced to NO_2_^−^ using nitrate reductase or other reductants before analysis. This method has a relatively low sensitivity with a detection limit of about 0.5 μM [55], but it can already detect NO with the level for biofilm dispersal and bacterial inhibition. Furthermore, low cost and simple operation are widely recognized advantages of this method. So Griess assay has been extensively utilized to detect NO release of various NO releasing materials for antibacterial application. For example, using Griess assay in solution, Boyer et al. detected the release of NO from polymeric nanoparticles with the concentration from 20–70 μM [56]. The NO releasing nanoparticles showed significant ability to disperse biofilms. However, this method cannot be used to detect intracellular NO, due to its relatively low sensitivity.

### 3.2. Fluorescence Method

Fluorescence method is a more sensitive method for NO detection. One of the widely used fluorescence NO detecting methods is DAN method. Similar to Griess assay, DAN method indirectly detects NO release by measuring NO_2_^−^ concentration. In acidic conditions, NO_2_^−^ reacts with 2,3-diaminonaphthalene (DAN) to form the fluorescent substance 1-(H)-naphthotriazole [57], which has high fluorescence efficiency under basic conditions (pH > 10) and can be quantitatively determined by fluorescence intensity at 450 nm after excitation with 365 nm light. DAN method is suitable for NO detection in solutions, which has a 50- to 100-times higher sensitivity than Griess assay [58], with detection limit of about 10 nM. Mosinger et al. detected the release of NO from NO releasing nanofibers [59]. However, this method requires excitation of UV light and an acid condition which are harmful to normal cells, so the use of DAN for NO detection in vivo is limited. Therefore, researchers tried to develop and design novel fluorescent probes which can be applied in vivo. Diaminofluoresceins (DAFs) can react with NO in the presence of oxygen under neutral conditions, resulting in the formation of a compound with strong green florescence when excited by visible light [60], representing a class of physiologically friendly NO fluorescent probes. Some of these probes have the characteristic of membrane permeability, which can monitor endogenous NO production in living cells [61,62], and have realized the imaging of intracellular NO in bacteria to study the physiological processes associated with NO [63,64].

### 3.3. Chemiluminescence Method

Chemiluminescence is another commonly used method for NO detection. The classical chemiluminescence method for NO detection is based on the reaction between ozone (O_3_) and NO to form excited state nitrogen dioxide (NO_2_*), which subsequently return to ground state accompanied by light [65]. The most widely used NO detecting system Sievers 280i Nitric Oxide Analyzer (NOA) is designed based on this principle, which has a high sensitivity with detection limit of up to 1 pM [66]. This commercially available NO detecting instrument is easy to operate and widely applied. For example, NOA is usually used for NO detection in the study of NO releasing indwelling medical devices [67,68,69]. Handa et al. detected NO release from NO releasing biomedical grade polymers [70]. Luminol method is another chemiluminescence method, which works in a way that NO can be rapidly oxidized by H_2_O_2_ to form ONOO^−^, and Luminol can be subsequently oxidized by ONOO^−^ accompanied by very strong light. 

### 3.4. Electrochemical Method

Different from the above three detecting methods which can only detect the cumulative release of NO over a period of time, electrochemical method can continuously measure the NO concentration in real time. Electrochemical oxidation is mainly used for NO detection, with the mechanism as follows: working electrode surface is modified with polymer film such as Nafion film [71] through which NO can pass at a rate proportional to its concentration. Once NO reaches the anode, it will be oxidized and produce a current proportional to its concentration. In 1990s, a commercial electrochemical NO microsensor ISO-NO meter was produced by World Precision Instrument (WPI) based on this principle. Since then, a variety of electrochemical NO sensors have been developed by this company, with the lowest detection limit of 0.2 nM [72]. NO microsensor can detect NO release in an intuitive and dynamic way, particularly suitable for the continuous detection of triggered NO release. For example, the NO releasing behavior of light-triggered NO donors can be evaluated. Using an ISO-NO meter, Sortino et al. monitored NO release triggered by light from NO photodonor embedded polymeric films [73]. Additionally, electrochemical NO sensors can be made in very small sizes to monitor the metabolic process of NO in tissues and cells without causing significant harms, which have been used to study real-time NO consumption related to hemoglobin activity in many pathogenic bacteria, such as *Escherichia coli* [74], *Mycobacterium bovis* [75], and *Pseudomonas aeruginosa* [76]. 

## 4. NO Donors for Antibacterial Application

In clinical practice, an appropriate concentration of NO is a key factor for antibacterial therapies. Another key factor is the duration of NO release in the physiological environment. NO has a relatively short half-life (less than 2s) in biological environments due to the radical nature of NO [77]. To achieve sustained release of NO for a long antibacterial time, proper storage and transportation tools are required. Researchers have developed and evaluated a variety of exogenous NO donors which can release NO in a controlled manner. Two types of NO-derived donors are mainly used in the antibacterial field: *N*-diazeniumdiolate (NONOate) [78,79] and *S*-nitrosothiol (RSNO) [80,81], which can store NO and release it in certain conditions. Under physiological conditions, one molecule of NONOate can spontaneously release two molecules of NO. However, RSNOs is much more stable than NONOate and release NO under the conditions of UV light [82], high temperature [83], metal ions [84], acids [85] or enzymes [86], and one molecule of RSNO can release one molecule of NO. More recently, 4-nitro-3-(trifluoromethyl)aniline and its derivatives [87] are used for NO donors in antibacterial application. Compared to NO donors mentioned above, they have a more controlled releasing mechanism, and can specifically release NO under the illumination of light. Compared with RSNOs which releases NO under UV light, this class of NO donors can release NO under visible light, which are more suitable for antibiofilm application, since visible light can penetrate the biofilm, but UV light cannot pass through the biofilm due to the UV absorber in the biofilm and strong absorptions of cellular components, especially DNA and hemes [88,89]. However, these visible light responsive NO donors have their drawbacks. For example, compared to the simple synthesis of NONOates and RSNOs, these light-triggered NO donors usually require a multistep synthesis [73,90].

Moreover, small molecular NO donors usually lack stability and specificity in vivo, resulting in quick and uncontrolled release of NO, thus limiting the therapeutic application. In order to meet the clinical requirements, the applications of pro-drug inspired researchers to develop polymeric NO donors as new antimicrobial agents with the methods of physical encapsulation and covalent conjugation. In addition, a combination of multiple antibacterial agents usually provides a better antibacterial effect. Thus, other antimicrobial moieties can be introduced into the NO releasing polymers, which shows more powerful synergistic bactericidal effects.

## 5. NO Releasing Polymeric Materials for Antibacterial Applications 

Polymer represents an efficient platform for NO loading. Polymeric NO donors formed by covalently conjugation or physically encapsulating small molecular-based NO donors to polymer platforms have proven to have excellent NO storage stability, prolonged NO release and optimized pharmacokinetics [38]. To date, various polymer-based NO releasing materials such as nanoparticles, nanofibers, coatings, and hydrogels have been prepared, which have shown great potential for antibacterial applications. Furthermore, some polymer-based NO releasing indwelling medical devices [68] have been developed to combat thrombus formation and bacterial infection.

NO releasing materials utilized for antibacterial application should meet the following requirements: (1) being easy to synthesize; (2) good stability during storage and application; (3) enough amount of NO release; (4) relatively long release time; (5) being able to fully contact bacteria. Herein we reviewed the relevant literature on the study of polymeric NO releasing materials with different NO donors for antibacterial applications, mainly focusing on their advantages such as targeting ability, stimulating responsiveness, anti-biofilm ability, anti-thrombosis ability, and biocompatibility.

### 5.1. NONOate Conjugated NO Releasing Polymeric Materials

**Figure 3:**
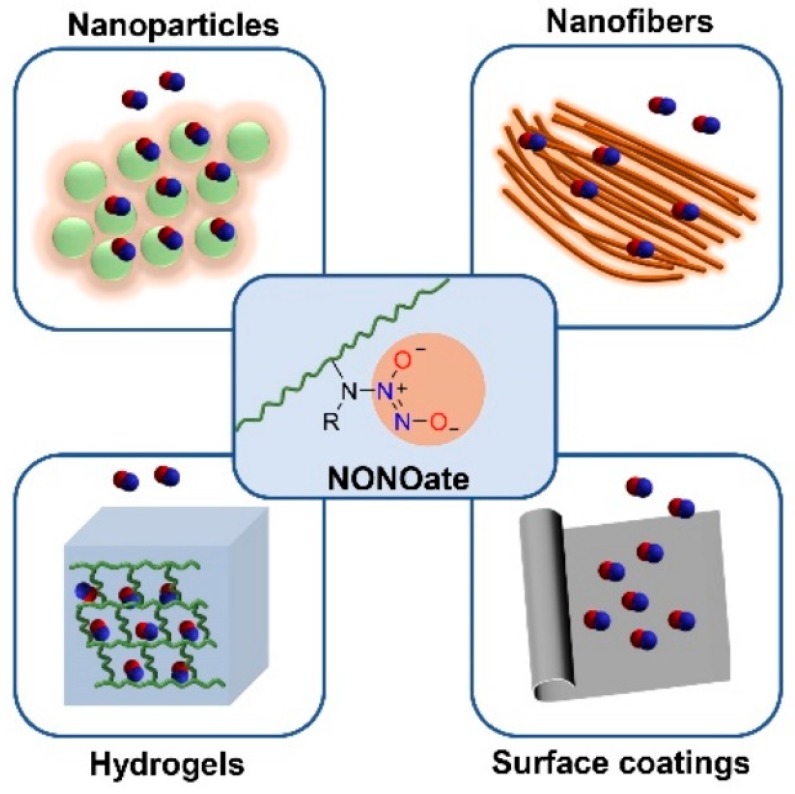
NONOate conjugated polymeric materials for antibacterial application: NO releasing hydrogels, reported in [95]; NO releasing nanoparticles, reported in [56,96,97]; NO releasing nanofibers, reported in [98]; NO releasing surface coatings, reported in [99,100,101,102].

*N*-diazeniumdiolates, also called NONOates, are compounds containing the functional groups [N(O)NO], which were firstly identified by Drago [91,92]. These functional groups are simply formed by reaction between secondary amines and high pressures of NO. NONOates were used prevalently to deliver exogenous NO for their facile synthetic procedure and excellent controlled release property [66]. Triggered by a proton source [93] such as humidity or acids, or a relatively high temperature [94], 1 mol of NONOates can release 2 mol of NO under physiological conditions spontaneously, making it an efficient scaffold for NO delivery. NONOates have a half-life that varies from a few seconds to several days, determined by the structure of secondary amine precursors, showing great potential in a variety of medical applications which require rapid or gradual production of NO. NONOates are conjugated onto various polymeric platforms, which have been utilized to prepare NO releasing materials such as hydrogels, nanoparticles, nanofibers and surface coatings for antibacterial applications (Figure 3). 

**Figure 4:**
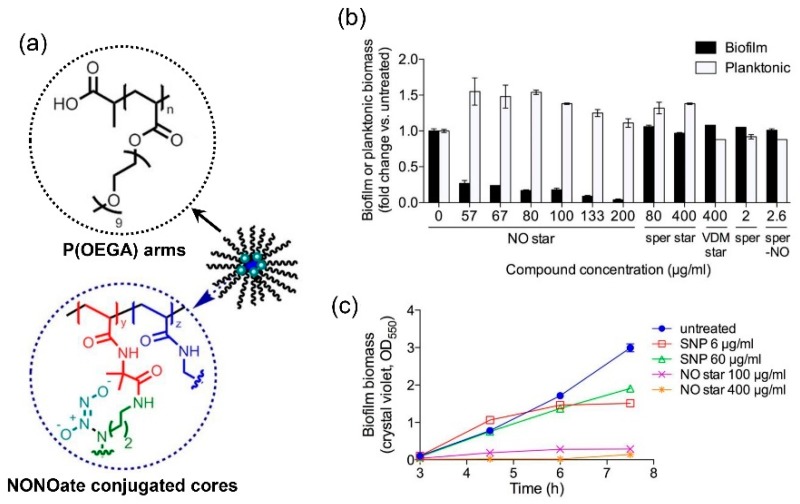
Structure and antibacterial property of NONOate conjugated star polymers. (**a**) The structure of NONOate conjugated star polymers, with P(OEGA) arms and NONOate conjugated cores. (**b**) Biofilm or planktonic biomass after treatment with NONOate conjugated star polymers, which showed dose-dependent prevention of biofilm formation. In contrast, the control groups, star polymers without NONOate conjugation, spermine and NONOate conjugated spermine showed no obvious preventing effect against biofilm formation. (**c**) Biofilm biomass after treatment of spontaneous NO donor sodium nitroprusside (SNP) and NONOate conjugated star polymers, by crystal violet staining (OD550). Reproduced with permission from [56]. Copyright 2014, American Chemical Society.

Polymer-based nanoparticles containing NONOate functional groups are extensively researched recently and have been proven to possess significant antibacterial and antibiofilm efficiency. Boyer et al. reported for the first time the use of star polymer for the encapsulation of NONOates [56] (Figure 4). They prepared a core cross-linked star polymer containing poly(oligoethylene methoxy acrylate) (P(OEGA)) arms and a cross-linked core, and encapsulated NONOate groups in the hydrophobic core of the star. This star polymer showed a rapid release of NO in the first hour and a sustained NO release in the following 70 h at pH 7.0, and cumulative release of NO was 60 μM when the concentration of star polymer was 1 mg/mL. This star polymer demonstrated great efficacy in preventing biofilm formation of *P. aeruginosa* with about 90% decrease of biofilm biomass when treated with 100 μg/mL NO star polymer and enhancing the dispersal of biofilms via a nontoxic mechanism over time and confine bacterial growth in a planktonic state.

In Boyer’s following researches, hybrid nanoparticles were synthesized by coating metal nanoparticles or metal oxide nanoparticles with NONOate functionalized polymers. These hybrid nanoparticles showed some new features. A polymer/gold hybrid nanoparticle called AuNP@P(OEGMA)-b-P(VBHA/NO) was prepared by grafting NONOate functionalized poly((oligoethyleneglycol methyl ether) methacrylate)-block-poly(vinyl benzyl chloride) onto Au nanoparticles (AuNPs) [103]. The hybrid nanoparticles showed higher stability and no burst release, with a slow and continuous NO release for 6 days. When treated with AuNP@P(OEGMA)-b-P(VBHA/NO), a significant reduction in biofilm bio-volume and increased biofilm dispersal of *P. aeruginosa* were shown compared to the untreated control. They also generated a NONOate functionalized polydopamine (PDA)-coated iron oxide nanoparticles (IONPs) called IONP@PDA-NO, and hydrophilic polymer P(OEGMA)-b-P(ABA) modified IONP@PDA-NO called IONP@PDA-HP-NO [104]. The cumulative NO released by IONP@PDA-NO is larger than IONP@PDA-HP-NO, resulting from the reduction of secondary amine after the conjugation of P(OEGMA)-b-P(ABA). Due to the hydrophilicity of P(OEGMA)-b-P(ABA), the release rate of IONP@PDA-HP-NO is more rapid than IONP@PDA-NO in aqueous solution. IONP@PDA-HP-NO showed great biofilm dispersal against *P. aeruginosa* with 79% reduction of biofilm biomass at a relatively low concentration of NO (0.375 × 10^−6^ M) compared to IONP@PDA-NO at the same NO concentration which did not stimulate the dispersal. This is probably due to the better colloidal stability of IONP@PDA-HP-NO, which penetrates the biofilm matrix more effectively. Furthermore, the magnetic property of IONP@PDA-HP was preserved, indicating its potential for magnetic targeted therapy.

In order to achieve good antibacterial efficiency, a relatively long NO releasing time is needed, especially on the surface of indwelling medical devices. To prolong the releasing time of NO, a superhydrophobic NO-releasing xerogel was prepared by Schoenfisch et al. by spray-coating a fluorinated silane/silica composite onto N-diazeniumdiolate-modified xerogel [100]. The material has the abilities of resisting bacterial adhesion and actively killing bacteria endowed by the superhydrophobic surface and inner NO-releasing xerogel. Furthermore, the duration of NO release was adjustable by varying the number of spray-coated superhydrophobic layers, and increasing layers from 0 to 12 extended the NO release duration from 59 to 105 h, demonstrating the potential of superhydrophobic topcoats to regulate release kinetics of drugs.

When applying NO releasing materials for antibacterial treatment, a large amount of NO release is probably needed. Thus, a platform that can store NO in a large volume should be prepared. Increasing the quantity of secondary amines on precursors has been proved to be an efficient measure to improve NO loading amount and antibacterial activities. Branched, hyperbranched [105,106] or dendrimer-like polymers containing many secondary amines are ideal platforms for NO loading. Wei et al. cross-linked branched polyethylenimine (bPEI) onto *N*-carboxy propionyl chitosan sodium (CPCS) to form CPCS-bPEI copolymer as a precursor to yield NONOates [107]. When the mole ratio of CPCS monomers to bPEI was 1:4, the overall amount of CPCS-bPEI-NO was 2.031 μmol/mg, much higher than NO donors reported before. CPCS-bPEI-NO exhibited excellent antibacterial activity against both Gram-negative *E. coli* and Gram-positive *Staphylococcus aureus* bacteria. In their following research, CS-PAMAM copolymer was synthesized by grafting poly(amidoamine) (PAMAM) dendrimers onto low molecular weight chitosan (CS) [108]. CS-PAMAM/NONOate formed by NO and CS-PAMAM precursor released NO totally for 1.7 μmoL/mg, and had a rapid initial release of NO in the first 2.5 h, providing a high instantaneous NO concentration to achieve an efficient antibacterial effect. Interestingly, CS-PAMAM itself has antibacterial activity due to its cationic nature endowed by its abundant secondary amines, which can strongly interact with the negatively charged cell membrane and disrupt the natural chemical balance inside the bacteria and consequently kill the bacteria. When NONOates are conjugated onto CS-PAMAM, the antibacterial activity is significantly enhanced. CS-PAMAM/NONOate demonstrated an excellent inhibitory effect on bacterial growth, mainly due to the high payload of NO. Furthermore, both NONOate functionalized CS derivatives have no significant cytotoxicity.

### 5.2. RSNO Conjugated NO Releasing Polymeric Materials

*S*-nitrosothiols, of which the general formula are RSNOs, are first synthesized in 1909. Such NO donors have good biocompatibility and are relatively more stable compared to *N*-diazeniumdiolates, but have a lower release rate. RSNOs can stimulate responsive decomposition and release NO, and is widely used in the modification of medical equipment against antithrombotic and antibacterial infections [109]. The most commonly employed RSNOs are *S*-nitrosoglutathione (GSNO) and *S*-nitroso-*N*-acetylpenicillamine (SNAP), which have diverse and remarkable biological effects. For instance, GSNO at low concentrations has been shown to afford significant protection to the ischemic myocardium [110] and SNAP is a potent vasodilator [111]. It is generally assumed that *S*-nitrosothiol is formed by substituting an H atom in a thiol group (–SH) on a compound with a nitroso group in a strongly acidic environment.

*S*-nitrosothiol based compounds do not spontaneously release NO. However, it can be rapidly decomposed and released by a catalytic reaction of UV light, heat, metal ions, ascorbic acid or an enzyme (such as superoxide dismutase) in vitro or in vivo to release a molecule of NO [112]. For example, Cu^2+^ is reduced to Cu^+^ by a thiolate, then Cu^+^ reacts with RSNO, and the sulfur–nitrogen bond cleaves to form disulfide and nitric oxide [113]. When RSNO is in a high temperature environment, the RS-NO bond is broken to form a molecule of NO and the corresponding disulfide bond. A molecule of NO is generated through ultraviolet light irradiation by inducing RS–NO bond rupture with ultraviolet light. At the same time, *S*-nitrosothiol reacts with the thiol radical generated by UV inducted homogenization to form another molecule NO. High concentrations of ascorbic acid or superoxide dismutase can act as a nucleophile to attack the RSNO directly and release NO. *S*-nitrosothiol is accompanied by some color changes during the release of NO. The characteristic color of the commonly used NO donor tertiary *S*-nitroso-*N*-acetylpenicillamine (SNAP) is green. Therefore, NO released from RSNOs can be observed by color, and more specifically, RSNO compounds can be detected at 225–261 nm, 330–350 nm, and 550–600 nm using an UV-vis spectrophotometer. 

**Figure 5:**
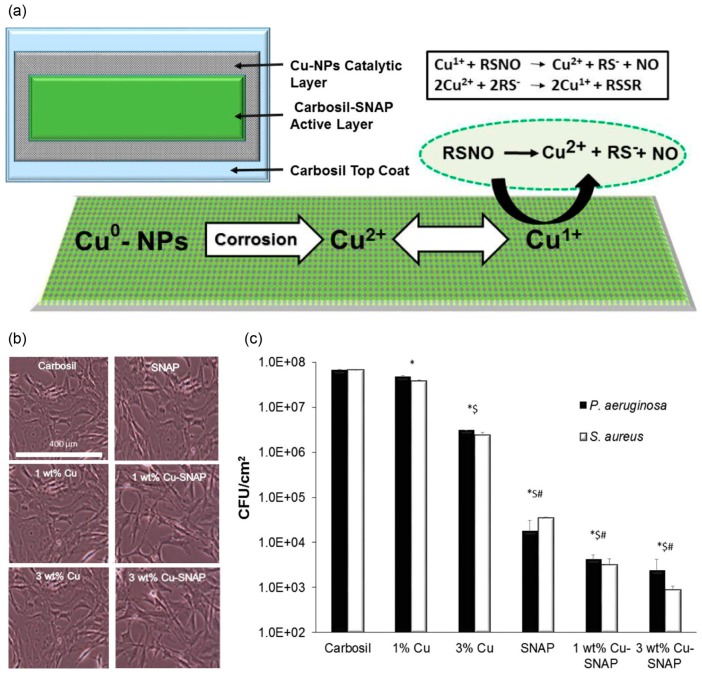
Structure, NO releasing mechanism, noncytotoxicity, reduced platelet adhesion, and bacterial inhibition of Cu-SNAP films. (**a**) Representative schematic of the polymeric composites with SNAP and Cu-NPs coating and the mechanism of NO releasling from SNAP and Cu-NP coating. (**b**) Optical images of 3T3 fibroblast cells after 24 h leachate treatment. (**c**) Inhibition of viable gram-negative (*P. aeruginosa*) and gram-positive (*S. aureus*) bacteria strains on the SNAP and Cu-NPs coating surface, in which *, $, and # indicate significant difference in CFU/cm^2^ of both bacteria compared to that of control, 1 wt % Cu, and 3 wt % Cu-Carbosil composites, respectively. Reproduced with permission from [116]. Copyright 2017, American Chemical Society.

It has been proven that the antibacterial effect of NO is dose-dependent [51,114]. SNAP modified polymers have been shown to release NO consistently for over 2 weeks, but NO concentration was near the lower end of physiological levels [67]. Therefore, increasing the release amount of NO is completely necessary. Researchers have designed catalytic release systems by combining SNAP with catalysts such as platinum nanoparticles [115], copper nanoparticles (Cu-NPs) [116], Cu_1.6_S nanoparticle [117], zinc oxide nanoparticles [118], ebselen [119], or selenium [120], to achieve a large amount release of NO. Handa et al. top-coated 1, 3, and 5 wt % of Cu-NPs to SNAP doped Carbosil films to fabricate Cu-SNAP films. The amount of NO released from Cu-SNAP films was observed to increase from 1.32 ± 0.6 × 10^−10^ mol min^−1^ cm^−2^ to 4.48 ± 0.5 × 10^−10^, 4.84 ± 0.3 × 10^−10^, and 11.7 ± 3.6 × 10^−10^ mol min^−1^ cm^−2^, respectively. The killing efficiency of bacteria and biofilms is greatly improved, and the amount of Cu leaching is significantly lower than the safety limit recommended by the FDA. Cytotoxicity assay showed that the Cu-SNAP combination was noncytotoxic to mammalian cells [116] (Figure 5). Meyerhoff et al. alternately assembled carboxyl-ebselen-fixed polyethyleneimine (e-PEI) and alginate (Alg) onto a substrate, followed by salt annealing and crosslinking to prepare a carboxyl-ebselen-based layer by layer (LbL) film. On the one hand, carboxy-ebselen is used to generate non-multiphase RSNO to accelerate NO production with a catalytic process. On the other hand, the reaction of ebselen with oxygen produces superoxide (O^2^•^−^), which inhibits the adhesion of bacteria to the surface of the material [119].

The release time of this NO donor in most biomedical applications is about 20 days. To prolong the release time of NO, Handa et al. covalently linked SNAP to poly(dimethylsiloxane) (PDMS) to form a highly stable NO releasing material. This strategy prevents leaching of the polymer matrix, avoids the generation of high NO bursts in a short period of time and the depletion of its NO reservoir. It achieved more than 125 days of sustainable NO release and bacterial inhibition and maintains long-term blood compatibility and biocompatibility [70]. Crystallization of SNAP is another way to prolong NO release. Meyerhoff et al. utilize a simple solvent impregnation technique to dope excess SNAP into the polymer. They found that the formation of SNAP crystals in the polymer phase achieved a long-term and stable supply of NO donors. The use of a suitable carrier can increase the release time of NO while maintaining a basic NO release rate [121]. Schwendeman and Meyerhoff et al. encapsulated SNAP into ester-capped polylactic acid-*co*-glycolic acid (PLGA) microspheres, and the release of NO lasted for more than 4 weeks. However, with uncapped PLGA, SNAP was slowly released for more than 10 days. Of course, copper ions, ascorbate and light are necessary conditions for decomposing NO donors to release NO, which can induce rapid release of NO by microspheres within a few hours under the action of light [122].

**Figure 6:**
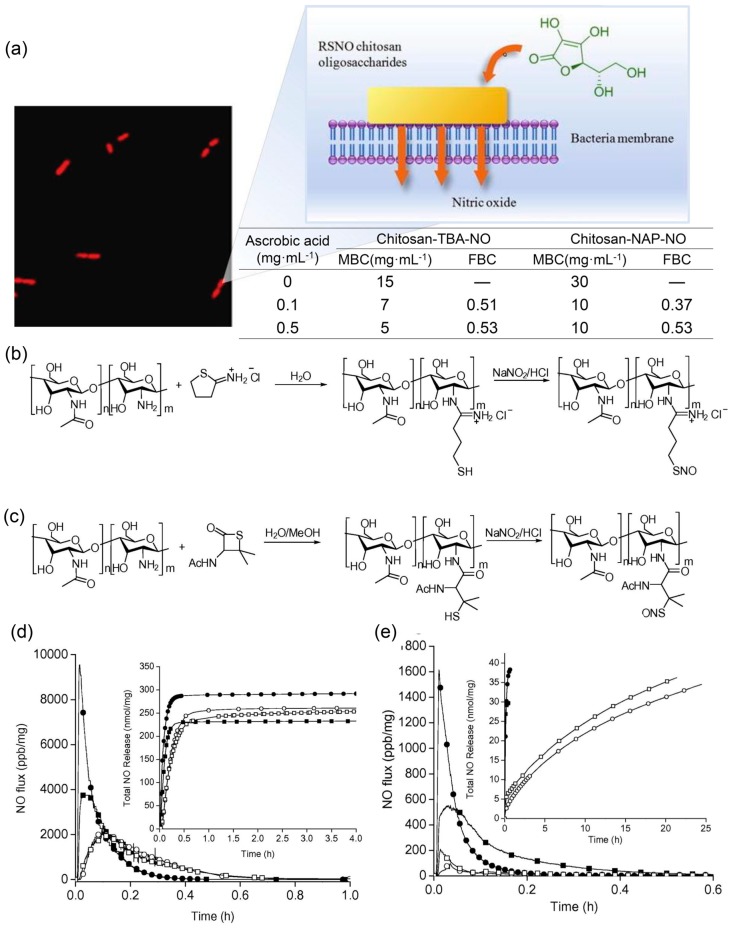
Synthesis of two *S*-nitrosothiol-modified chitosan oligosaccharides: chitosan-NAP-NO and chitosan-TBA-NO, and their release of NO and antibacterial effect in ascorbic acid. (**a**) The antibacterial effect of chitosan-NAP-NO and chitosan-TBA-NO respectively. (**b**) Synthetic method of chitosan-TBA-NO. (**c**) Synthetic method of chitosan-NAP-NO. NO release properties for (**d**) chitosan-NAP-NO and (**e**) chitosan-TBA-NO in 0.1 (square) and 0.5 (circle) mg mL^−1^ ascorbic acid with (open) and without (solid) DTPA. Inset: total NO release with time (nmol mg^−1^). Reproduced with permission from [125]. Copyright 2015, Elsevier.

RSNO is often used in combination with other antibacterial agents to synergistically kill bacteria. Brocchi et al. used NO donors to coat catechin-reduced silver nanoparticles (AgNPs) with mercaptosuccinic acid (MSA). The synergistic sterilization of NO and nano-Ag particles greatly reduced the minimum inhibitory concentration (MIC), which was 62, 125, and 3 μg/mL for AgNPs-catechin, AgNPs-catechin-MSA, and AgNPs-catechin-*S*-nitroso-MSA-incubated *P. aeruginosa* (American Type Culture Collection, ATCC 27853, Gaithersburg, MD, USA) [123]. They also incorporated NO donor *S*-nitrosoglutathione (GSNO) into a thermoresponsive Pluronic F-127 (PL)-chitosan (CS) hydrogel. It was desirable to produce a therapeutic amount of NO in a controlled spatial and temporal manner while co-sterilizing with chitosan. The test found that the MIC value decreased to 0.5 μg·mL^−1^ relative to the hydrogel without GSNO doping. Cytotoxicity experiments showed that PL/CS hydrogels containing GSNO were not toxic to Vero cells at concentrations below 13.23 μg·mL^−1^ [124]. Schoenfisch et al. also modified chitosan by synthesizing two kinds of *S*-nitrosothiol modified chitosan oligosaccharides (Figure 6). They compared the NO release curves of these two chitosans catalyzed respectively by copper ion solution, light and ascorbic acid. Although the amount of NO released by chitosan-NAP-NO is greater than that of chitosan-TBA-NO, chitosan-TBA-NO bactericidal is better than chitosan-NAP-NO due to cations. Under the catalysis of ascorbic acid, chitosan-TBA-NO caused a reduction in the survival rate of *P. aeruginosa* by four orders of magnitude. Significantly reduced cell death for chitosan-TBA-NO was observed compared to chitosan-NAP-NO, as a result of excessive NO release for oxidative damage to normal cells [125].

### 5.3. Visible Light Responsive NO Releasing Polymeric Materials

Given that NO can be therapeutic or toxic depending on its concentration, an accurate control of dosage of NO release is essential in clinical treatment [126]. However, traditional NO donors like NONOates are not very controllable, which usually release NO spontaneously under physiological conditions. As an external stimulus, light is a powerful tool for triggering chemical reactions in biological environment with rapidity and accuracy without affecting important physiological parameters such as temperature, pH, and ionic strength [87]. Hence, photoresponsive NO donors were designed by several researchers such as *S*-nitrosothiols (RSNOs), photoactive metal nitrosyls compounds, and nitrobenzene derivatives. 

**Figure 7:**
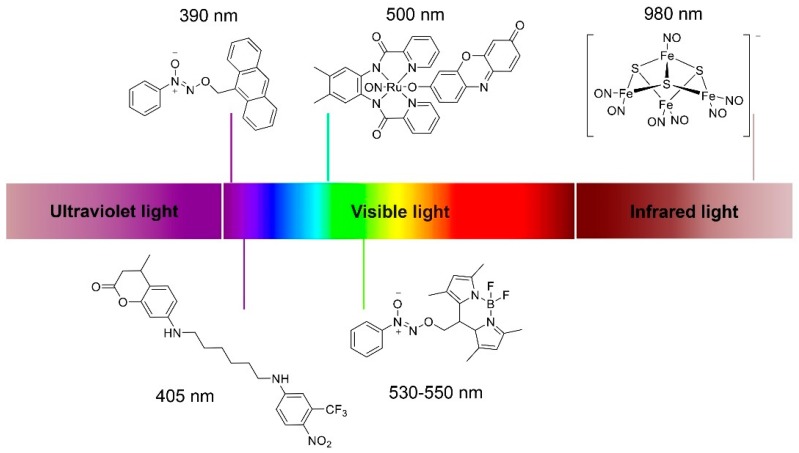
Chemical structures of small molecule photoresponsive NO donors triggered by visible light or near-infrared light.

As mentioned above, *S*-nitrosothiols (RSNOs) can release NO triggered by UV light, representing one of the most common classes of NO photoresponsive NO donors. However, UV light cannot penetrate biofilms very well mainly due to the UV absorbing compounds in extracellular matrix secreted by microorganisms [88], which may affect the antibacterial properties of UV-triggered NO donors. Furthermore, UV can be harmful to normal cells of human, so NO releasing materials triggered by physiological friendly visible lights or near-infrared lights (NIR) would have more advantages in antibacterial therapy. Researchers have developed small molecule photoresponsive NO donors triggered by visible lights with wavelengths of 390 nm [127] (also reported with 380 nm [128]), 405 nm [73], 500 nm [129], 530–550 nm [130], and even NIR with wavelengths of 800 nm [131] and 980 nm [132] (partly presented in Figure 7). 

Photoactive metal nitrosyls compounds are constructed by coordinating metal centers (M) such as Ru [133], Fe, or Mn [134] with NO and suitable multidentate ligands. Triggered by light, these compounds can release NO, mainly due to the electronic transitions from dπ (M)-NO(π*) bonding orbitals to dπ (M)-NO(π*) antibonding orbitals and subsequent dissociation of M-NO bonds [135]. By altering multidentate ligands [136] or adding a strongly colored dye molecule as another ligand [129], the photosensitivity of metal nitrosyls compounds to visible light would be enhanced, thereby releasing NO under visible light. However, such compounds have not been conjugated onto polymeric systems or widely investigated for antibacterial applications, probably due to the complex synthetic path and potential toxicity of metal ions to normal cells.

**Figure 8:**
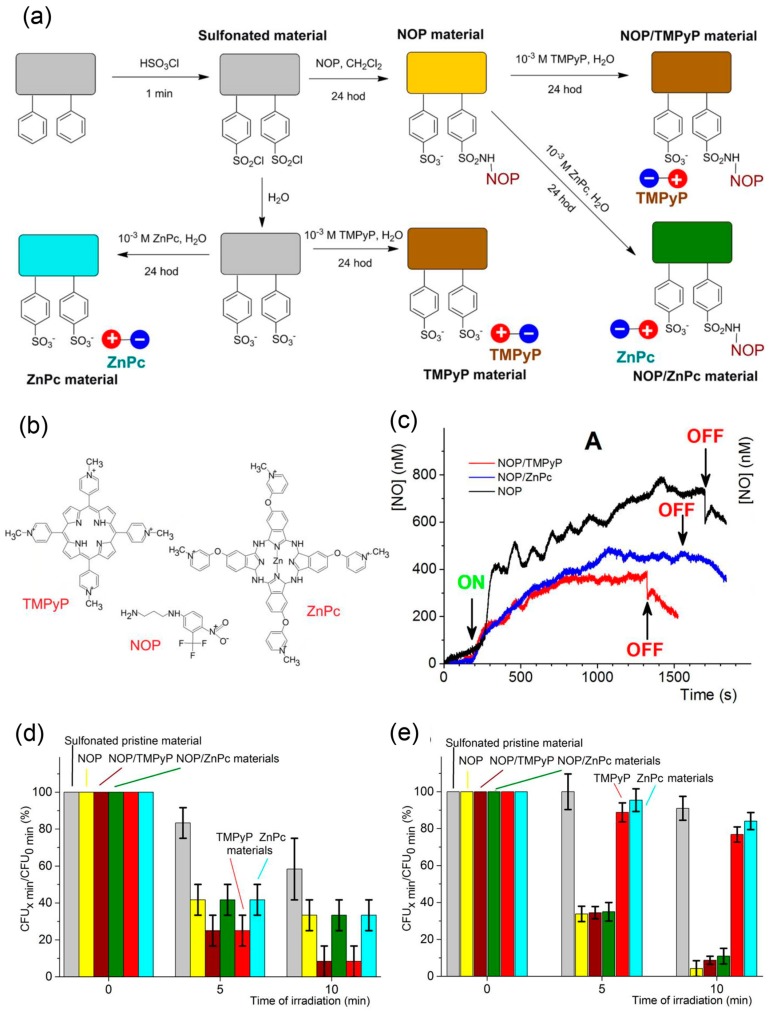
Preparation, NO releasing behavior, and antibacterial properties of photoactive nanofiber materials. (**a**) Schematic diagram of the preparation protocol for the photoactive nanofiber materials. (**b**) Structures of the photoactive compounds used in this work: NO photodonor (NOP), tetracationic TMPyP, and tetracationic ZnPc photosensitizers. (**c**) Amperometric detection of the NO photoreleased from the surface of NOP (black trace), NOP/TMPyP (red trace), and NOP/ZnPc(blue trace) materials. (**d**) Surface photoantibacterial activity of NOP, NOP/TMPyP, NOP/ZnPc, TMPyP, and ZnPc materials against *E. coli* compared with sulfonated pristine nanofiber material. (**e**) Space photoantibacterial activity of NOP, NOP/TMPyP, NOP/ZnPc, TMPyP, and ZnPc materials against *E. coli* compared with sulfonated pristine nanofiber material. Reproduced with permission from [59]. Copyright 2015, American Chemical Society.

Recently, metal-free visible light responsive NO donors have been investigated by researchers. Photoresponsive nitrobenzene derivatives are the most representative metal-free photo-controlled NO releasing donors. The main types of these compounds are 4-nitro-3-(trifluoromethyl)aniline and its derivatives, which are usually called NO photodonors (NOPDs or NOPs) [90]. Their light-triggered NO releasing mechanism is described as below: the steric hindrance of the ortho CF_3_ substituent leads to twisted geometry and non-conjugated state of nitro group with respect to the aromatic plane, resulting in an overlap between the p orbital of oxygen atom of nitro group and the adjacent p orbital of the aromatic ring in both ground and excited states [137]. When exposed to light, the nitro group can easily rearrange to nitrite, and subsequently rupture the O-N bond to generate NO [87,137,138]. The Sortino group studied the antibacterial property of these compounds recently. Nanofiber materials capable of releasing NO and singlet oxygen (^1^O_2_) under visible light have been prepared by this team by conjugating NO photodonors and ^1^O_2_ photosensitizers TMPyP or ZnPc onto sulfonated polystyrene nanofibers, specifically called NOP, NOP/TMPyP, NOP/ZnPc, and ZnPc materials [59] (Figure 8). All of the prepared materials containing NOP release NO under visible light, but NO release of NOP/TMPyP and NOP/ZnPc materials is decreased compared to NOP material mainly due to the overlaps between absorption bands of NOPs and ^1^O_2_ photosensitizers and the resulting shielding effects. NOP/TMPyP and NOP/ZnPc materials which can release NO and ^1^O_2_ showed stronger surface bacterial inhibition on *E. coli* than NOP materials which can only release NO, demonstrating the synergistic antibacterial effect of NO and ^1^O_2_. However, NOP/TMPyP and NOP/ZnPc materials showed slight reduction in space antibacterial effect compared to NOP materials, since ^1^O_2_ has a short lifetime and diffusion pathway, which would have less impact on bacteria than NO with longer lifetime and diffusion pathway. Besides attaching NOP and ^1^O_2_ photosensitizer onto different sites, molecular hybrids with NOP and ^1^O_2_ photosensitive units such as porphyrin [139] or boron dipyrromethene (BODIPY) [90] derivatives were designed and synthesized by this team. These molecular hybrids can release ^1^O_2_ and NO under visible lights, providing a synergistic strategy of NO and photodynamic therapy (PDT). Recently, they synthesized a fluorescence reporting NO photo-releasing molecular hybrid by covalently connecting NOP with coumarin, then embedded it into poly(lactic-*co*-glycolic acid) (PLGA) to form an antibacterial polymeric film [73]. This film can generate NO under visible light with concomitant fluorescence reporting, and demonstrated significant antibacterial activity against *E. coli*. Both NO release and bacterial reduction were displayed only under illumination. Additionally, molecular hybrids containing 4-nitro-3-(trifluoromethyl)aniline moiety and another moiety with the ability to target specific sites would realize targeting NO release [140]. Besides 4-nitro-3-(trifluoromethyl)aniline-based NO photoreleaser, a novel green-light-induced NO photoreleaser containing a cupferron unit and a BODIPY derivative unit was recently synthesized by them [130]. The compound is stable in the dark but generates NO under visible green light. Though it has not been utilized for antibacterial now, it shows potential as an antimicrobial agent controlled by unharmful green light.

**Figure 9:**
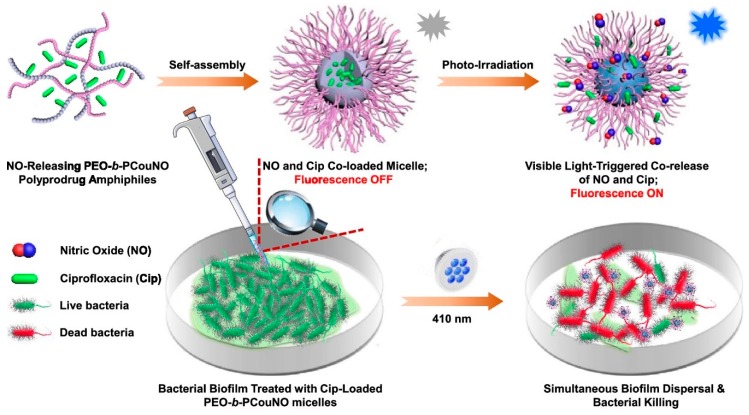
Fabrication of visible light responsive micelles, and the co-release of NO and Cip under irradiation of 410 nm light to concurrently disperse biofilms and kill bacteria. Reproduced with permission from [146]. Copyright 2019, American Chemical Society.

Since biofilm-associated infections pose greater threat to our society, its necessary to develop visible light responsive NO donors capable of combating biofilms. However, there are no reports on 4-nitro-3-(trifluoromethyl)aniline-based NO photoreleaser for antibiofilm application. *N*-nitrosoamine-based NO donors are also photo-triggered NO donors and several small molecular *N*-nitrosoamine-based NO donors have been explored [141,142,143,144,145]. More recently, Hu et al. reported a NO releasing micelle bearing visible light responsive NO donors [146]. Unlike the conventional preparation of NO releasing polymers which usually need postmodification approaches, controlled chain length of *N*-nitrosoamine-based NO releasing monomers were polymerized by utilizing RAFT polymerization with a polyethylene glycol modified chain transfer agent. The resultant amphiphilic polymers were self-assembled into micelles in aqueous solution, which can release NO under 410 nm light irradiation with a remarkable fluorescent report and demonstrated the ability of biofilm dispersal against *Pseudomonas aeruginosa*. When loading hydrophobic antibiotics, ciprofloxacin (Cip), into the cores, NO and Cip can be released simultaneously under 410 nm light to efficiently eradicate bacterial biofilms and kill bacteria (Figure 9). 

## 6. Anti-Biofilm Properties of NO

Biofilms are aggregates of microorganisms encased in a self-produced matrix of extracellular polymeric substance (EPS) and can form on almost any moist surface [147]. Bacterial biofilms are highly resistant to host immune defenses and conventional antibacterial agents such as antibiotics mainly due to the protection of the EPS matrix [148] (Figure 10①). Once formed on living tissues or indwelling medical devices, biofilms are extremely difficult to eradicate by antimicrobial treatments, which results in chronic and recurrent infections [149,150]. The biofilm-related infections have a considerable impact on patient health and present many clinical challenges.

The life cycle of biofilms contains attachment, colonization, maturing and dispersal [109], and these processes are regulated by an intracellular second messenger cyclicdiguanylate-guanosine monophosphate (c-di-GMP) (Figure 10①,②). In general, biofilm formation is enhanced as the intracellular concentration of c-di-GMP increases, while more bacteria enter planktonic mode as the intracellular concentration of c-di-GMP decreases [152]. The mechanism of this regulation by c-di-GMP has not been fully elucidated, maybe c-di-GMP achieves this by activating the enzymes related to biofilm formation. NO has been found to be an important signaling molecule in regulating these processes [153]. During the late development stage of biofilm life cycle, NO is synthesized endogenously and activates bacterial phosphodiesterase to degrade c-di-GMP, thus promoting biofilm dispersal [152]. Biofilms dispersal can also be induced by exogenous NO at low and non-toxic doses [154], different from the killing effect of NO with high concentration.

Three main strategies to target biofilm-associated bacterial infections so far have been classified by Boyer [152] et al. according to different development stages of biofilms, which are (i) preventing microbial adhesion and biofilm formation, (ii) targeting established biofilms, and (iii) targeting persisters. NO can play a role in all three methods. Before biofilm formation, the primary task of a surface (especially used for medical devices) is to inhibit microbial adhesion, thus preventing biofilm formation. Super-hydrophobic polymer coated surfaces [100] and low-fouling polymer coated surfaces [99] have been prepared by several researchers which showed prevention of bacterial adhesion to some extent, and modifying these surfaces with NO releasing functional groups can effectively enhance inhibition of bacterial adhesion. Once biofilms are formed, NO releasing nanoparticles [56,103,104] can serve as antimicrobial agents to disperse biofilms and kill bacteria and are usually more efficient than conventional antibacterial agents. Persisters represent a small fraction of the biofilm community which are tolerant of conventional antibiotic treatments, thus can be re-established after treatment. Once persisiters have emerged, the main measure to effectively treat these repeated biofilm infections is to combine them with various antibacterial agents. It has been proved that biofilm cells will regain their susceptibility to antibiotics when returning to the planktonic state (Figure 10③) [153]. Therefore, NO can synergistically enhance the bactericidal effect of antibiotics. Boyer et al. prepared a polymeric nanoparticle which can release NO and gentamicin simultaneously, demonstrating synergistic effects [96]. Specifically, NO disperses biofilm into planktonic state and gentamicin kills the bacteria. The co-releasing nanoparticle can eradicate *P. aeruginosa* in a minimum concentration of 10 μM equivalent to gentamicin, while a minimum concentration of 100 μM gentamicin alone was needed to eradicate *P. aeruginosa*. Since microbial cell membranes are usually negatively charged due to negatively charged membrane proteins or phospholipids on their surface [147], polycationic polymers which contain negatively charged groups—such as primary and secondary amines, quaternary ammonium, and quaternary phosphonium—can incorporate with microbial cell membranes and disrupt the natural chemical balance inside the bacteria and consequently kill them. It has been reported that NO is combined with this kind of antimicrobial polymers in some research, which shows the antibiofilm effect is enhanced in a synergistic way [155,156].

## 7. NO Releasing Polymers Applied in Indwelling Medical Devices

With the extensive application of polymeric materials, almost all indwelling biomedical devices are made with polymers these days, including intravascular catheters, urinary catheters, indwelling blood pumps, vascular assist devices, orthopedic implants, and other indwelling biomedical devices [157]. However, these indwelling medical devices usually face great challenges in long-term clinical applications because of the thrombus formation, bacterial infection, innate immune response, and other device-related complications, which may result from plasma protein adsorption, bacterial adhesion, and biofilm formation on biomaterial surfaces, causing not only health threats but also extra treatment costs to patients. In recent years, antimicrobial coating materials have been developed for biomedical devices to combat device-associated infections. Biomedical devices with silver or antibiotic doped coatings are clinically tested and have a dominant position in the current market [158]. However, silver has potential toxicity to patients, and long-term use of antibiotics can easily cause bacterial resistance. Furthermore, these two antimicrobial agents cannot inhibit the formation of thrombi, which limit the further application of these biomedical devices, especially in blood-associated applications. Therefore, the development of novel biomedical devices with surfaces capable of preventing bacterial adhesion and thrombus formation is urgently needed in clinic. NO releasing materials may be ideal candidates for the development of novel biomedical devices which can effectively prevent bacterial infection and thrombus formation, since NO is identified to be capable of preventing platelet activation and adhesion, and inhibiting bacterial proliferation and biofilm formation [109]. *S*-Nitroso-*N*-acetylpenicillamine (SNAP), a commonly used nitrosothiol typed NO donor, has been extensively investigated to incorporate into biomedical grade polymers to create NO releasing materials [159,160,161,162,163] because of its low cost, safety, stability during release and storage, demonstrating the potential for long-term applications [164]. Meyerhoff and Handa et al. prepared NO releasing catheters by doping SNAP into the Elast-Eon E2As polymers [67]. These SNAP-doped catheters showed significant reduction of thrombus area (from 5.06 ± 1.44 to 1.56 ± 0.76 cm^2^) and bacterial adhesion (approximately 90%) during the 7-day implantation in sheep veins. Due to the excellent antibacterial and antithrombotic properties of the SNAP-doped materials, a series of following studies were conducted by these researchers. Furthermore, many researches have shown that some special polymeric surfaces are capable of inhibiting bacterial adhesion and thrombus formation due to the inherent properties of these polymers or the ordered surface nanostructures. Research has started to focus on the synergistic effects of NO donors and these special surfaces.

Fluorinated polymers such as polytetrafluoroethylene (PTFE) and polyvinylidene fluoride (PVDF) are widely used to prepare various biomedical implants and devices because of their excellent mechanical properties, great thermal stability, high chemical inertness, and improved biocompatibility [165,166]. Owing to the highly hydrophobic surface property, fluorinated polymers show inherent anti-inflammatory and thromboresistant properties [167]. However, these properties are limited, and further biomedical applications need enhanced antibacterial and antithrombotic properties. Doping NO donors into these fluorinated polymers seems to be a good solution, but poor compatibility between them usually results in a significant amount of chemical leaching [167]. Meyerhoff et al. synthesized a fluorinated SNAP derivative C_2_F_5_-SNAP as NO donor and PDVF tubing was swollen in this C_2_F_5_-SNAP solution to prepare C_2_F_5_-SNAP doped PDVF tubing [168]. Due to the fluorous–fluorous interactions between C_2_F_5_-SNAP and PVDF polymer, a relatively low leaching of C_2_F_5_-SNAP was observed, which was lower than 10% after 11 days. In their following studies, a new fluorinated SNAP derivative DiCF_3_Bn-SNAP was synthesized and doped into poly(vinylidene fluoride-*co*-hexafluoropropylene) copolymer (PVDF-HFP). This NO releasing fluorinated polymer showed a very low leaching of DiCF_3_Bn-SNAP which was only 0.6%. Both of the fluorinated SNAP derivative doped fluorinated polymers discussed above showed significant antimicrobial and antibiofilm activities against both Gram-positive *S. aureus* and Gram-negative *P. aeruginosa* strains.

Some hydrophilic surfaces made by hydrophilic polymers such as polyethylene glycol (PEG) or polyzwitterions have shown excellent antifouling properties, which would suppress the nonspecific adsorption of proteins, thus preventing thrombus formation, innate immune response, and bacterial infection [169]. These hydrophilic surfaces can form hydration layers when in contact with water, which can act as a barrier to prevent the adsorption of proteins [170]. Due to the tight electrostatic interaction with water molecules, the hydration layers formed by polyzwitterions is denser and thicker than PEG, so zwitterionic materials are superior to PEG-based materials in preventing biological foulants and more biocompatible [171]. Recent research showed the synergistic antimicrobial and antifouling properties of the combined application of NO releasing polymers and polyzwitterion coatings. Handa et al. covalently grafted antifouling zwitterionic terpolymer BPMPC onto SNAP doped biomedical grade copolymer CarboSil [172]. This NO releasing polymer top-coated with antifouling polyzwitterions showed higher NO release and reduced SNAP leaching compared to uncoated sample, and a significant reduction of protein adhesion (about 84–93%) and viable bacteria adhesion (about 99.1%) were observed. Recently, naturally produced antifouling surfaces have attracted great interest because of their excellent biocompatibility. Handa et al. fabricated a NO releasing polymer coated with a hydrophilic surface formed by the self-assembly of naturally-produced hydrophobins [173]. This hydrophobin-coated NO releasing polymer showed enhanced protein-resistant, bactericidal, antiplatelet and cytocompatible properties, indicating a possibility of future application in medical device coatings.

**Figure 11:**
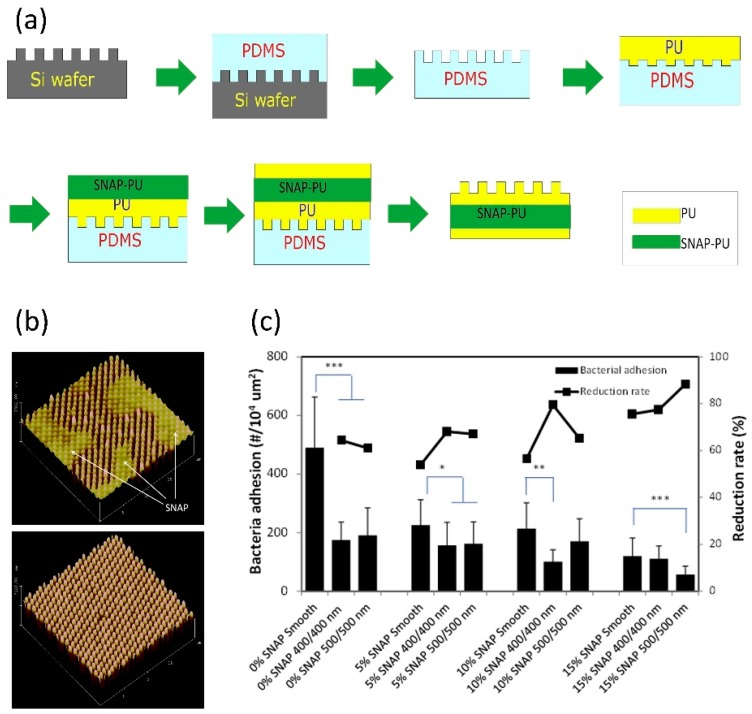
(**a**) The preparation of SNAP-doped textured PU surface *using* soft lithography two-stage replication molding technique. (**b**) 3D atomic force microscope (AFM) images of NO releasing textured polyurethane film surface, in which thin top layer showing the diffusion of SNAP onto top surface (above), and thick top layer showing normal textured surface feature (below). (**c**) Bacterial adhesion and reduction rates (against smooth regular PU polymer) on NO releasing textured polyurethane surfaces, in which asterisks represent a statistically significant difference in bacterial adhesion relative to non-textured surface, with * denotin *p* < 0.05, ** denoting *p* < 0.01, and *** denoting *p* < 0.001. Reproduced with permission from [157]. Copyright 2017, Elsevier.

Inspired by natural antifouling surfaces—such as shark skin [174], shell [175], or lotus leaf [176]—researchers have developed textured surface with similar micro- or nano-size topographic features. Such surface modification can reduce the surface contact area and change the surface energy, and the designed biomimetic surfaces have been identified to be effective in reducing bacterial adhesion and biofilm formation. Recently, research has begun to focus on the synergetic effects of surface texturing and NO release to inhibit bacterial adhesion and biofilm formation. Meyerhoff et al. designed and prepared a “sandwich-like” polymer film bearing a top surface layer with ordered pillar topographies and a NO releasing sublayer made with SNAP-doped CarboSil 20 80A silicone-polycarbonate-urethane (PU) (Figure 11) [157]. The top surface layer was fabricated with a soft lithography two-stage replication molding technique and the pillars of pattern on the top surface have the submicron dimensions. This SNAP-doped textured PU surface with sublayer containing 15 wt % SNAP showed inhibition against biofilm formation of *S. epidermidis* for up to 28 days. Interestingly, the texturing seemed to be more effective in inhibiting bacterial growth and bacterial formation under shear conditions than static conditions. 

In summary, thrombus formation and bacterial infection are the main drawbacks of indwelling biomedical devices for long term use. The combined application of NO releasing material and specific surfaces made by fluorinated polymers or polyzwitterions or modified with micro- or nano-size topographic features showed great potential in enhancing the antibacterial property of biomedical devices. However, antithrombotic property of these materials needs further research, strict in-vivo experiments and clinical tests are also needed before clinical applications.

## 8. Conclusions and Prospects

In this review, we highlighted the latest advances in NO releasing polymeric materials for the prevention and treatment of bacterial infections. Although NO has been shown to be effective in inhibiting bacterial biofilms, its direct use in clinical settings is still limited due to its short half-life and instability. To overcome this challenge, polymeric materials with different NO donors have been developed for antibacterial application. Among them, NONOates and RSNOs conjugated polymers are easy to synthesize, which makes them the two most widely used NO releasing polymers. NONOates have the advantages of a large amount of NO release and a variety of polymeric platforms available for conjugation in different applications. RSNOs can release NO upon various stimulations, and are more stable under physiological conditions, thus giving a more sustained NO release, which makes it more suitable for preparing biomedical devices capable of combating thrombus and infections. Visible light responsive NO releasing polymers can release NO triggered by physiologically friendly and biofilm-permeable visible light, representing a smarter and more precise tool for combating bacterial infections at specific locations especially those related to biofilms. In summary, what is required of NO releasing polymeric materials in the future is not only a sufficient release amount and prolonged release time, but also precise targeting ability and controlled release time and dose. Furthermore, NO releasing polymers can synergistically enhance the antibacterial effect of other antibacterial agents such as antibiotics, polycations, or silver nanoparticles, showing an effective antibacterial therapy that combines NO releasing polymers with other antibacterial agents. 

We also reviewed the NO-releasing indwelling medical devices, which demonstrated great antibacterial and antithrombotic properties during long-term applications. Therefore, the NO releasing antibacterial strategy can be a potential direction for combating infectious diseases related to indwelling medical devices. More importantly, functions and properties of biomedical devices need a revolutionary progress to meet the requirement of clinical therapies in the future. In recent years, flexible wearable devices have become a focus of biomedical studies. Flexible wearable devices have shown great potential in the diagnosis and treatment of diseases, which will profoundly change the life of mankind in the next several years. However, the potential bacterial infection caused by long-term use of flexible wearable devices have not been heeded, but it would have great limitation in biomedical applications without the antibacterial strategy. Since NO has less impact on the electrical and mechanical properties of devices, and has lower cytotoxicity to normal human cells human compared to conventional antibacterial agents, NO releasing materials can be recognized as an ideal antibacterial strategy applied on flexible wearable devices. Besides antibacterial properties, NO may play a bigger role on flexible wearable devices. For example, flexible wearable devices integrating NO sensors can monitor the health status of human related to regulation of NO. Furthermore, flexible wearable devices with NO releasing moieties may have the function of tissue regeneration. In summary, NO releasing materials applied on flexible wearable devices would have multifunctional benefits for human health.

## Figures and Tables

**Figure 10:**
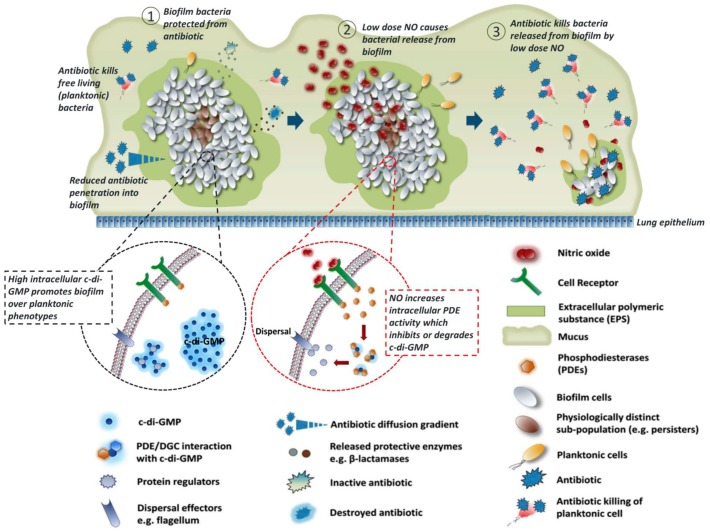
Disruption of biofilm antibiotic tolerance regulated by NO. (**①**) The formation of biofilms due to high concentration of c-di-GMP, which endowed bacteria antibiotic tolerance. (**②**)Biofilm dispersal regulated by low dose of NO, since NO reduce the intracellular levels of c-di-GMP. (**③**) Plankronic bacteria releasing from biofilm restore sensitivity to antibiotics and are killed by antibiotics. Reproduced with permission from [151]. Copyright 2017, Elsevier.
